# Application of Plackett–Burman Design in Screening of Natural Antioxidants Suitable for Anchovy Oil

**DOI:** 10.3390/antiox8120627

**Published:** 2019-12-06

**Authors:** Yun-Qi Wen, Chang-Hu Xue, Li-Li Xu, Xiao-Han Wang, Shi-Jie Bi, Qian-Qian Xue, Tao Zhang, Yong Xue, Zhao-Jie Li, Gui-Dong Chen, Xiao-Ming Jiang

**Affiliations:** 1 College of Food Science and Engineering, Ocean University of China, Qingdao 266003, China; wenyq0715@163.com (Y.-Q.W.); xulili8339@163.com (L.-L.X.); wangxh0527@163.com (X.-H.W.); xue851668452@163.com (Q.-Q.X.);; 2 College of Food Science and Technology, Hebei Agricultural University, Baoding 071000, China

**Keywords:** fish oil, natural antioxidants, screening, Plackett-Burman design

## Abstract

Considering the safety of synthetic antioxidants, more and more natural antioxidants have been developed and utilized in foods. This study aimed to screen out a natural antioxidant combination from many antioxidants, which could significantly affect the oxidation stability of anchovy oil, while Plackett–Burman design (PBD) methodology was employed in this screening. According to the statistical results of this design, sesamol, dihydromyricetin, teapolyphenol, and rosemary acid were four significant parameters on the oxidation stability of anchovy oil. Moreover, dihydromyricetin presented the best antioxidant effect among nine kinds of selected antioxidants when they were used alone in anchovy oil. Meanwhile, a combination including sesamol (0.02%), teapolyphenol (0.02%). and rosemary acid (0.02%) was adopted, and its antioxidant ability was similar to that of tert-butylhydroquinone (TBHQ). Additionally, phytic acid as a synergist was used and combined with sesamol, and the antioxidant ability of this combination was better than that of TBHQ. This study presented a reference for the industrial applications of natural antioxidants and synergists in anchovy oil.

## 1. Introduction

Docosahexaenoic acid and eicosapentaenoic acid (DHA/EPA) supplementation during the critical period of early life development show better outcomes in respect to visual and cognitive parameters of the offspring. Fish and algae oils are important sources of DHA and EPA, and generally suitable for incorporation into foods. However, their applications are limited as the formation of an undesirable taste and odour. These unpleasant sensory are caused by secondary oxidation products of unsaturated fatty acids, and the oxidation of EPA or DHA esters are five to eight times faster than the oxidation of ethyl linolenate [1,2,3].

Auto-oxidation of unsaturated fats in oil includes three steps as follows: during the initiation step, unsaturated fatty acids generate free radicals (R**•**) by losing a hydrogen atom under the catalysis of metal ions and the action of external energy. During the propagation step, unsaturated fatty acid free radicals (R**•**) reacts very fast with molecular oxygen and turns into peroxide radicals (ROO**•**). The peroxyl radical can abstract a hydrogen atom from another unsaturated fatty acid, and then form a hydroperoxide (ROOH) and a new free alkyl radical (R**•**). Hydroperoxides (ROOH) are the primary oxidation products. Lipid hydroperoxides are tasteless and odorless. However, in the presence of heat, metal ions, etc., they will decompose to secondary oxidation products (aldehydes, ketones, alcohols, hydrocarbons, volatile organic acids, and epoxy compounds), and many of which contribute off-odors and off-tastes. During the termination step, the produced radicals can be terminated when two radical species combine to form a non-radical species, such as oxidized polar/non-polar dimers or trimers of lipids [4,5].

The application of antioxidants in fish oil is one of the most common methods to inhibited lipid oxidation. Antioxidants can donate hydrogen atoms to radicals, resulting in the interruption of free radical propagations, and then the antioxidant free radicals form a stable peroxy-antioxidant compound [6,7]. Bivalent transition metal ions, Fe^2+^ especially, can catalyze lipid antioxidants [8]. Some compounds can form an iron chelate, which can inhibit iron-catalyzed hydroxyl radical formation and lipid peroxidation [5]. These compounds have an auxiliary antioxidant effect and are generally known as synergists, such as phytic acid, citric acid, and ethylene diamine tetra acetic acid (EDTA). Antioxidants include natural antioxidants and synthetic antioxidants. Synthetic antioxidants like tert-butylhydroquinone (TBHQ) have raised concerns about their safety. These negative effects and perceptions lead to the growing demands of natural ingredients. Many previous studies have proved the natural additives as better antioxidants and thermally stable as compared to synthetic antioxidants in different edible oils [9,10,11].

Many kinds of natural antioxidants have been found and extracted, such as dihydromyricetin, tea polyphenol, ferulic acid, ascorbic acid, sesamol, lycopene, rosemary acid, and carnosic acid. The combined applications of natural antioxidants have been widely adopted as the combination of antioxidants usually has better antioxidant effect than their application alone. However, it is should be mentioned that some of combinations present no synergistic effect of antioxidant activity. Dietary synthetic antioxidants together with α-tocopherol were effective in sparing tocopherols oxidation and preventing the oxidation of fresh frozen chicken patties. However, the combinations of natural tocopherols, green tea and rosemary did not reveal synergistic effects [12]. The antioxidant effectiveness of natural antioxidants is also influenced by food components, such as the inherent characteristics (ionic strength and pH) of the food, the food matrix (emulsion, foam, aqueous, and protein), and ingredients [4]. Thus, when many antioxidants can be selected and used in combination for different specific food materials, it is necessary to screen the optimal natural antioxidants, and identify the antioxidants that show significantly positive effects on oxidation stability of lipids.

Nine kinds of antioxidants were prepared in this experiment. As some antioxidants might not show significant antioxidant effectiveness in fish oil, it was necessary to screen them out. Then, the other antioxidants presenting significant antioxidant effectiveness could be selected and combined for applying to fish oil. The antioxidant effectiveness of natural antioxidants could be influenced accidentally by various levels of factors, such as food components, solubility of antioxidants in the matrix, the interaction between different kinds of antioxidants, etc., which meant that the process of screening and application of the antioxidants in food was cumbersome. Plackett-Burman design (PBD) is a design method which uses the first order polynomial equation to rapidly and effectively screen out the most important factors from multiple variables at one time approach [13]. Therefore, PBD could be tried to use in the screening of antioxidants to simplify the screening process. All these nine kinds of natural antioxidants would be added to the fish oil at the same time according to PBD. The antioxidant effectiveness of each antioxidant would be simultaneously investigated at each approach. Finally, the information on whether the antioxidant effectiveness of each antioxidant was significant and the sorting of antioxidant effectiveness of these antioxidants would be presented. The aim of this study was to screen antioxidants from nine natural antioxidants for their combined application suitable for anchovy oil. Furthermore, the synergists including phytic acid and citric acid, were compared of their abilities in enhancing the oxidation stability of anchovy oil.

## 2. Materials and Methods

### 2.1. Materials and Reagents

Potassium hydroxide (KOH) used for neutralization and the nitric acid (65%, *v*/*v*, GR grade) were obtained from Merck Chemicals Co., Ltd. (Shanghai, China). Activated clay and Notit-8015 activated charcoal used for decoloration were purchased from Jiejingclay Co., Ltd. (Leping, Jiangxi, China) and Zhongji Chemicals Import & Export Co., Ltd. (Shanghai, China), respectively. Deionized water was produced by a Millipore Milli-Q water purification system (Burlington, MA, USA). Na_2_S_2_O_3_-solution (0.100 moL/L) and *p*-anisidine were purchased from Shanghai Yuanye Biotechnology Co., Ltd. (Shanghai, China). All other reagents of analytical grade were obtained from Sinopharm Chemical Reagent Co., Ltd. (Qingdao, China). Antioxidants including dihydromyricetin (DM, purity ≥ 98%), teapolyphenol (TP, purity ≥ 90%), ferulic acid (FA, purity ≥ 98%), ascorbic acid (VC, purity ≥ 99%), sesamol (SO, purity ≥ 90%), lycopene(LE, purity ≥ 10%) and TBHQ (purity ≥ 98%) were all purchased from Qingdao Mobio Biotech Co.,Ltd. (Qingdao, China). Rosemary acid (RA, purity ≥ 5%) and carnosic acid (CA, purity ≥ 20%) were purchased from Shaanxi Huike Plant Development Co., Ltd. (Xi’an, Shanxi, China). Ascorbyl palmitate (AP, purity ≥ 98%) were purchased from Shanghai Yuanye Biotechnology Co., Ltd. (Shanghai, China). Synergist including citric acid (purity ≥9 9%) and phytic acid (purity ≥ 90%) were obtained from Qingdao Mobio Biotech Co.,Ltd. (Qingdao, China).

### 2.2. Preparation of Fish Oil

Crude anchovy oil was purchased from Zhoushan Xinnuojia Bioengineering Co., Ltd. (Zhoushan, China) before adding antioxidants. The chemical refining of crude anchovy oil was conducted according to the method previously reported by Yuxiang [14]. The refined fish oil presented the following major fatty acids as measured by gas chromatography (GC) according to Lingyu [15]: C14: 0–7.7%, C16: 0–27.2%, C16: 1–8.2%, C18: 0–5.4%, C18: 1ω9–29.5%, C18: 2ω6–1.6%, C18: 3ω3–1.1%, C20: 1–0.9%, C20: 4ω6–1.0%, C20: 5ω3–5.8%, C22: 2–0.5% and C22: 6ω3–11.0%. The determination of metal ion content in refined anchovy oil referenced Zhipeng [16]: Cr: 0.207 mg/kg, Fe: 2.700 mg/kg, Cu: 0.140 mg/kg, Zn: 6.478 mg/kg, As: 0.008 mg/kg, Cd: 0.002 mg/kg, Pb: 0.010 mg/kg.

### 2.3. Storage of Anchovy Oil Supplemented with Natural Antioxidants

The natural antioxidants were prepared by grinding to fine powder in mortar. Antioxidants with poor fat solubility were dissolved in 100% ethanol and then added to the prepared fish oil. Fat-soluble antioxidants were added directly into fish oil and the same volume of ethanol was also added [17]. The mixture of anchovy and antioxidant was stirred for 10–15 min at room temperature, and then was divided in sterilized 20 mL brown glass bottles, which were subsequently put into a 60 °C oven, respectively. The samples were taken out on setting days for testing peroxide value and anisidine value.

### 2.4. Peroxide Value and Anisidine Value

The peroxide value (POV) of fish oil was determined according to the AOCS method Cd 8b-90 [18] with some modification. Briefly, about 2 g of anchovy oil was weighed into an iodine flask. 30 mL mixed solvent and 1 mL saturated potassium iodide solution were added successively. After the reaction, 100 mL distilled water was added before titration with 0.0100 mol/L Na_2_S_2_O_3_-solution. When the solution was titrated yellowish, 3 mL 0.2% starch solution to visualize the end-point.

The *p*-anisidine value (*p*-AV) was determined according to the AOCS method Cd 18-90 [18]. The total oxidation value (TOTOX) of fish oil could comprehensively reflect the content of primary and secondary oxidation products of fish oil. TOTOX was calculated by the POV and *p*-AV of fish oil with the following Equation (1) [19]:

TOTOX value = 2POV + *p*-AV (1)

### 2.5. Oxidation Kinetics of Anchovy Oil

The oxidation kinetics of lipids can be calculated using the following Equation (2) [20]:

(2) $$\frac{dY}{dt}=-k\;Y^{n}$$

where *Y* is the concentration of the peroxides, *t* stands for time, *k* stands for the reaction rate constant, and *n* stands for the order of the reaction. Oxidation of lipids follows a first-order reaction (*n* = 1) [21,22,23]. Therefore, integrating Equation (3) shows the following equations:

ln*Y* = ln*Y*_0_ + *kt* (3)

where *Y*_0_ stands for the initial concentration of oxidation products.

Plots of ln*Y* versus *t* produce the slope (*k*) of the linear regression line to fit the data points. In the initial stage of fish oil oxidation, the rate of hydroperoxide formation was higher than its decomposition rate, resulting in a gradual increase in the hydroperoxide content (an increase in the POV). At this stage, the change of POV with time followed Equation (3). *k* as the oxidation rate constant could reflect the speed of oxidation of fish oil. Antioxidants could affect the speed of oxidation. Therefore, the *k* value could reflect the protective effects of antioxidants on the fish oil. In the later stage of oxidation, the rate of hydroperoxide formation was less than its decomposition rate, resulting in a gradual decrease in hydroperoxide content and a decrease in POV.

### 2.6. Screening of Natural Antioxidants by PBD

PBD was employed to screen seven kinds of natural antioxidants that significantly (*p* < 0.05) affected oxidative stability of fish oil. These seven kinds of natural antioxidants were set as seven variables (from X1 to X7). As the number of experiments for PBD needed to be a multiple of four, 12 experiments were designed. The unassigned variables including X8, X9, X10, and X11 were set as dummy factors for being used to estimate the experimental errors. Each factor was tested at two levels of high level (+1) and low level (−1) and all factors, their levels were shown in the Table 1. For the PBD experimental results, the effects of variables were evaluated by the first-order polynomial equation as follows [24]:

(4) $$Y=\beta_{0}+\sum_{i=1}^{k}\beta_{i}X_{i}\;(i=1,\ldots ,k)$$

where, *Y* stands for response, *β*_0_ stands for intercept, *β_i_* the regression coefficients, *X_i_* stands for the level of the independent factor. The *k* value mentioned in 2.6 section was selected as *Y*. These factors which significantly (*p* < 0.05) affected the *Y* were identified according to their *F*-value.

### 2.7. Application of Synergist and Screened Natural Antioxidant

Citric acid and phytic acid were two common synergists, which were separately combined with the antioxidant sesamol and then added to the produced anchovy oil for the storage experiments described in Section 2.3. Then, their antioxidant properties were compared with the synthetic antioxidant TBHQ. The antioxidants screened by PBD were also applied to fish oil. Sesamol, teapolyphenol and rosemary acid were selected for storage experiments. The above experimental designs were summarized in Table 2.

### 2.8. Statistical Analysis

All experiments were carried out in triplicate, and the data were expressed as mean ± standard deviation (*n* = 3). PBD were generated by Design-Expert 8.0 software (Stat-Ease Inc., Minneapolis, MN, USA). The statistical analysis of experimental data was performed by SPSS 20.0 software (SPSS Inc, Chicago, IL, USA). The mean values of measured parameters were compared with one-way ANOVA, followed by Dunnett’s test. All statistical tests were taken to be significant at *p*-values less of 0.05.

## 3. Results and Discussion

### 3.1. Effect of Nine Antioxidants Used Alone on the Oxidation Stability of Anchovy Oil

**Figure 1:**
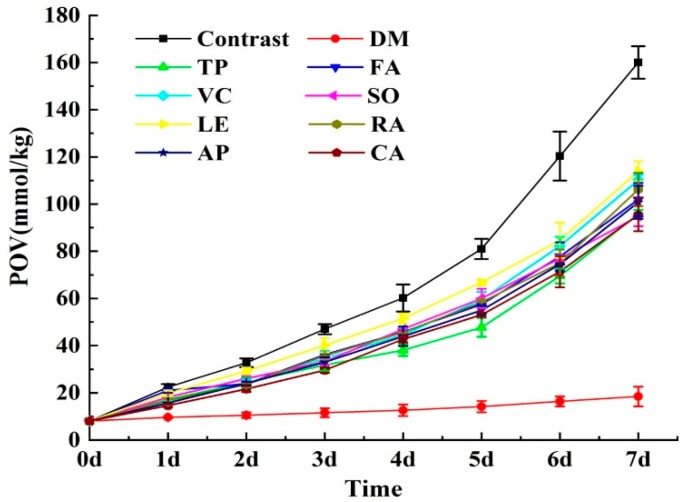
Peroxide value change of anchovy oils containing natural antioxidants during the storage experiment at 60 °C. Among them, the contrast was anchovy oil without antioxidant. Data represent means ± S.D. for *n* = 3.

Nine prepared antioxidants with an amount of 0.04% (w%) were added to the produced fish oils, respectively. The mixtures were separately put into a 60 °C oven for seven days. Figure 1 showed the POV change of fish oils during the storage. The POV of the fish oil without antioxidants (contrast) increased the fastest, increasing to 160.05 mmol/kg within seven days, which was 19.91 times higher of the initial value. The POV of the fish oil added with DM increased the slowest, increasing to 18.45 mmol/kg within seven days, which was only 2.29 times higher than the initial value. The POV of the fish oil added with SO, CA, TP, AP, FA, RA, VC, and LE correspondingly reached 94.83, 95.54, 95.74, 100.78, 101.91, 106.24, 110.12, and 113.91 mmol/kg, which was 11.79, 11.88, 11.90, 12.53, 12.67, 13.21, 13.69, and 14.16 times higher of the initial value, respectively. During the initial stage of storage, the POV of all samples increased slowly, and it was in the initiation phase of oil oxidation. During the late stage of storage, the POV of all samples increased fast, and it was in the propagation phase of oil oxidation.

The TOTOX change of fish oils during the storage was shown in Table 3. During the storage period, the TOTOX of fish oil without antioxidant was significantly higher than the fish oil added with antioxidants (*p* < 0.05), and the TOTOX reached 690.59 after 7 days. The TOTOX of fish oil with DM was significantly lower than other fish oil samples (*p* < 0.05), and the TOTOX finally reached 92.28. The TOTOX of all fish oil samples gradually increased, and their values were as follows after seven days: DM < CA < SO < TP < AP < FA < RA < VC < LE < Contrast.

Plots of ln (POV) versus storage time produced the linear regression line to fit the data points acquired from seven days storage experiment. The coefficients of determination (R^2^) and oxidation rate constants (*k*) of the linear regression line of fish oils oxidation kinetics were also produced as shown in Table 4. The R^2^ of ten equations range from 0.9906 to 0.9972 indicating that the fitted model explained 99.06–99.72% of the observation variability around the mean, and the oxidation kinetics of fish oil selected followed the first-order reaction rate equation. The *k* was obtained from a comprehensive evaluation of all POV from the seven days storage, and was more suitable for reflecting the oxidative stability of fish oil than a single POV. The *k* of all fish oil samples showed as follows: *k* (DM) < *k* (FA) < *k*(TP) < *k* (SO) < *k* (LE) < *k* (RA) < *k* (AP) < *k* (CA) < *k* (VC) < *k* (Contrast).

These results obtained from Figure 1, Table 3 and Table 4 suggested that all fish oil samples added with antioxidants had better oxidative stability than that of fish oils without antioxidants. The antioxidant ability of fish oil added with DM was the best according to the three indicators (POV, TOTOX, *k*), while the VC was not good at all these three indicators. The color of fish oil changed from light yellow to red when LE was used, indicating LE had a great influence on the exterior quality of fish oil. Besides, the antioxidant ability of LE was not significant in fish oil sample. Therefore, VC and LE were not considered in later PBD experiment.

DM was a natural flavanonol compound found in the human diet such as fruits, vegetables, and teas, while it possessed numerous bioactivities, such as antioxidant, antiinflammation, anticancer, antitussive, antibacterial, antithrombotic, and antitumor activities, and so on [25,26]. The antioxidant ability of cookie fortified with dihydromyricetin was significantly improved compared with untreated cookie. Meanwhile, DM showed the strongest effect to suppressed lipid and protein oxidation among four selected flavonoids [27]. Another important finding was that DM showed strong DPPH scavenging ability and high ORAC value in vitro and in culture hepatic cells [28]. DM had more antioxidant activity than RA through exhibiting antioxidant effects on DPPH, ABTS, hydroxyl, or superoxide free radical scavenging activity in vitro [29]. Ye et al. [30] reported that DM was also more potent than BHA in the prevention of soybean oil oxidation. Zuo et al. [29] reported that phenolic hydroxyl was indispensable to the antioxidant activity of flavonoids and the number and location of phenolic hydroxyl of the flavonoids were expected to the different antioxidant activities.

SO was a major lignan isolated from sesame seeds and sesame oil, and possessed biological actions such as inhibition of lipid peroxidation, enhancement of radical scavenging [31]. SO was better than RA in enhancing the oxidation stability of anchovy oil during the seven days storage. The result was in line with the previous study. SO (0.13 w%) performed better than the rosemary extract (containing 0.13 w% phenolic diterpenes) in suppressing POV and headspace volatiles compounds during storage of fish oil for 14 days [32]. CA possessed beneficial effects on health including antioxidative, anti-inflammatory, antibacterial, anti-cancer, and neuroprotective properties. Besides, Lin et al. showed that CA could be absorbed by the *Caenorhabditis elegans* (*C. elegans*) and enhanced the health span [33]. CA was used in the extraction process of anthocyanins and flavonols for inhibiting oxidation. The experimental results showed that CA played an effective antioxidant role in the extraction process with a green and safety guarantee [34]. FA showed wide variety of biological activities such as antioxidant, anti-inflammatory, antimicrobial, antiallergic, hepatoprotective, anticarcinogenic, and antithrombotic [35]. Grain bran, whole grain foods, citrus fruits, bamboo shoots, coffee, etc., were the rich sources of FA. Additionally, herbs and spices were the other predominant sources of FA. The antioxidant potential of FA was attributed to the formation of a resonance stabilized phenoxy radical because of the presence of a phenolic nucleus and an extended side chain conjugation [36]. VC was commonly used in the food industry for its antioxidant capacity and providing an additional source of VC. VC was water solubility compound. The antioxidant property of VC was limited, although VC was uniformly dispersed in fish oil under the action of ethanol. The antioxidant property of AP was better than that of VC as AP had good oil solubility. However, the antioxidant property of AP was not outstanding in this study. LE was an unsaturated fat-soluble carotenoid, which quenched the damaging free radicals, especially the singlet oxygen. It had gained great interest as its biological properties in the antioxidant activity, anti-inflammatory, cancer prevention, and cardiovascular protection. It was abundant in tomatoes and tomato-based products [37,38]. The addition of LE significantly increased linseed oil antioxidant capacity (ABTS test). 80 mg/kg of LE led to the same induction time at 110 °C obtained using 200 mg/kg of the antioxidant BHT. However, significant differences were presented between LE extract-enriched and pure linseed oil for color [39].

### 3.2. Application of PBD in the Screening of Natural Antioxidants for the Combined Utilization

The PBD was used to screen for antioxidants that had a significant effect on the oxidative stability of fish oil. Seven natural antioxidants, including DM, SO, CA, TP, AP, FA, and RA, were added together to the fish oil. PBD matrix of twelve runs and the experimental values of oxidation rate constants were shown in Table S1. Oxidation rate constants (*k*) were calculated by Equation (3) and results were showed in Table S2. ANOVA was implemented for evaluating the effect of each parameter (natural antioxidant). The results were shown in Table S3. According to statistical results, the *F* value was 6.99, indicating the significance of the selected model. The relative importance of these seven parameters was presented as follows: F > A > B > E > D > G > C. Meanwhile, it should be revealed that F (SO), A (DM), B (TP) and E (RA) played key roles in inhibiting the oxidation of fish oil.

**Figure 2:**
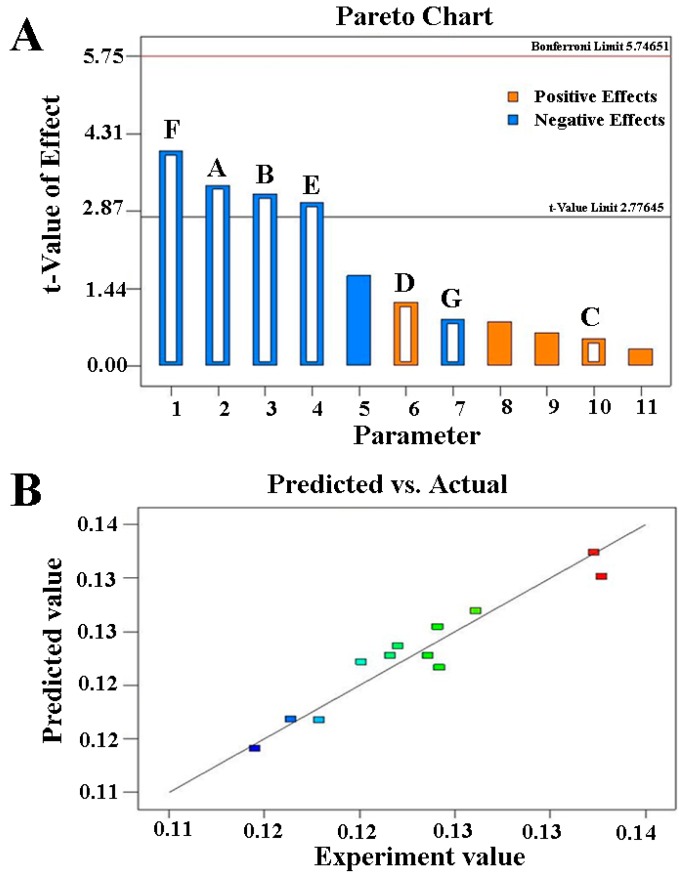
(**A**): The Pareto chart for the effect of the factors on the *k*. Factors with blue and orange indicating a negative and positive effects on the *k*, respectively. A: DM (dihydromyricetin), B: TP (teapolyphenol), C: CA (carnosic acid), D: AP (ascorbyl palmitate), E: RA (rosemary acid), F: SO (sesamol), G: FA (ferulic acid); (**B**): Predicted value versus experimental value of *k*. Points with different colors indicating different *k* values.

The Pareto chart showed the ordered standardized effect of each parameter and it could identify the magnitude and the importance of each parameter. The standardized effect of parameter would be statistically significant, as long as it exceeded a threshold. Meanwhile, the most important effects were ranked in a decreasing order according to their significance. As shown in Figure 2A, X6: SO (F), X1: DM (A), X2: TP (B), and X5: RA (E) showed significant effects (*p* < 0.05, 0.0163, 0.0285, 0.0331, and 0.0385, respectively) on the *k*. The mathematical model of the linear regression analysis for the *k* in terms of actual factors were established according to these results, which were as follows:

*Y*(*k*) = 0.1512 − 0.4950DM − 0.2358TP + 0.0375CA + 0.1750AP − 0.2242RA − 0.2942SO − 0.1283FA (5)

It was obvious that SO, DM, TP, and RA showed negative effects on the *Y*(*k*). The regression coefficient obtained for *Y*(*k*) was 0.9245, indicating that the fitted model could explain 92.45% of the observation variability around the mean. The prediction of *Y*(*k*) was compared with the experimental *Y*(*k*) as shown in Figure 2B. Both *Y*(*k*) showed a close agreement, further indicating the suitability of the mathematical regression model.

**Figure 3:**
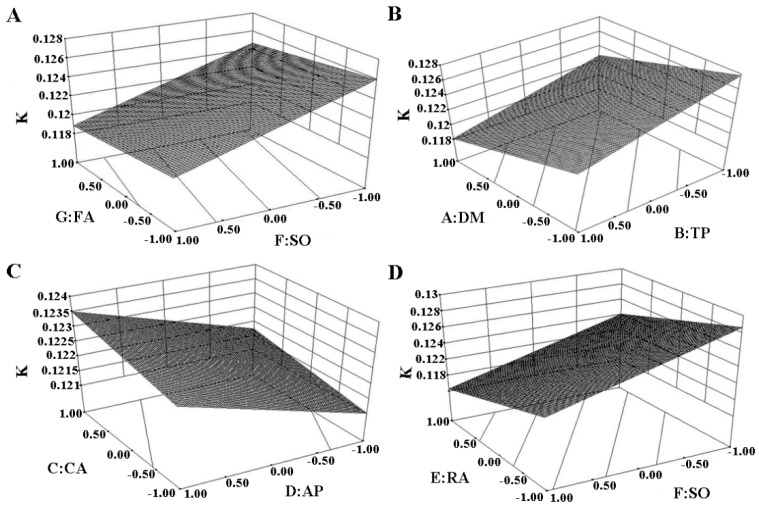
Three-dimensional (3D) response surface graphs for the effect of natural antioxidants on the k. (**A**): The interactive effects of FA (ferulic acid) and SO (sesamol); (**B**): The interactive effects of DM (dihydromyricetin) and TP (teapolyphenol); (**C**): The interactive effects of CA (carnosic acid) and AP (ascorbyl palmitate); (**D**): The interactive effects of RA (rosemary acid) and SO (sesamol).

Three-dimensional (3D) response surface graphs for the effect of natural antioxidants on the *k* according to the magnitude of each parameter effect on the certain response were shown in Figure 3. Figure 3A provided that an increase of SO led to a significant decrease in the *k* when the amount of FA added was constant. While, the effect of the amount of FA addition on *k* was not significant. Figure 3B showed that an increase of DM or TP all led to a significant decrease in the *k*. Figure 3C showed that an increment in the CA or AP all resulted in a little increase in the *k* without significant correlation. The RA increase could also significantly lead to the increase of *k* as shown in Figure 3D.

The antioxidant property of natural antioxidants combination could be superior to the individual antioxidant effects. A synergistic co-antioxidant could regenerate the antioxidant by donating hydrogen. VC and ascorbyl ester was a well-functioning antioxidant pair for α-tocopherol as the regeneration of active tocopherol from its oxidized radical counterpart [7]. The synergistic antioxidant effects among lycopene, vitamin E, vitamin C, and β-carotene was determined. The antioxidant ability of their combination was substantially superior to the sum of the individual antioxidant effects. Meanwhile, these interactions were benefit to the antioxidant effectiveness of natural antioxidants [38]. The study of antioxidants used in brown ales showed that flavonoids, total phenolics, and nonflavonoid phenolics, extracted from both the malt and the hops, presented synergistic effect of antioxidant activity [40]. The effect of *Moringa oleifera* leaves extract on the oxidative stability of soybean oil was studied under 12 days accelerated storage. The mixture of 0.1% *Moringa oleifera* leaves extract and 0.02% α-tocopherol displayed a significant synergistic effect [41]. F (SO), A (DM), B (TP), and E (RA) could significantly suppress the oxidation of fish oil according to the results of PBD in this study. Therefore, these four antioxidants could present synergistic effect of antioxidant activity.

**Figure 4:**
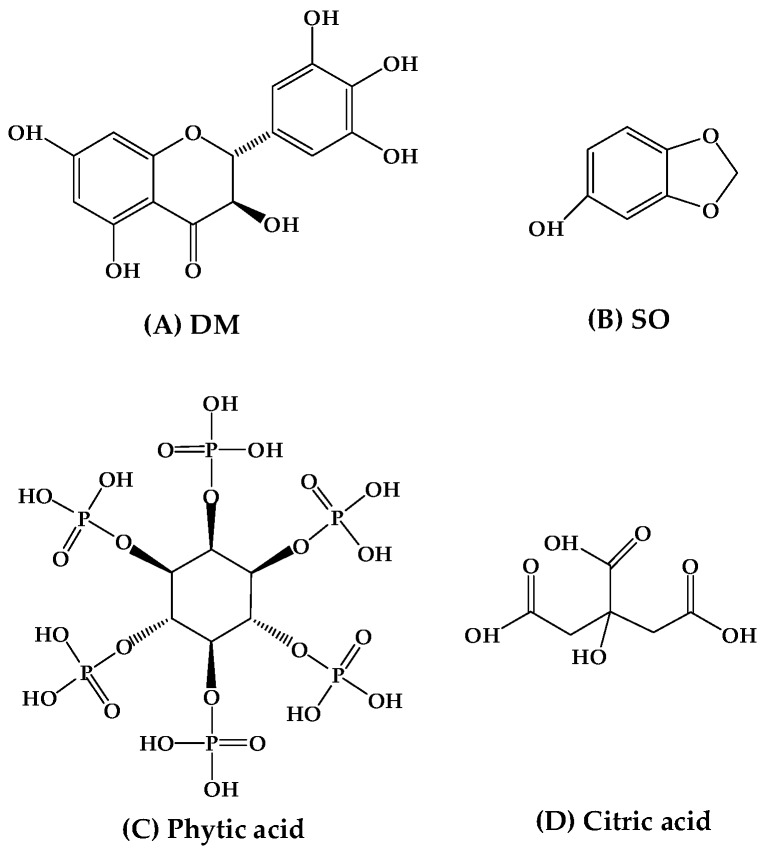
Molecular structure of antioxidants/ synergists: (**A**) DM; (**B**) phytic acid; (**C**) SO, and (**D**) citric acid.

From the above results, the relative importance of DM was lower than SO. However, the antioxidant efficacy of DM was better than that of SO when they were used alone (Section 3.1). This might be related to their solubility in fish oil. The synergistic effect of antioxidants appeared to be based in part on the decreasing potential of the various antioxidants and on their ability to transform the free radicals of antioxidant to their native structure [38]. SO was more soluble in fish oil than DM, leading that it was more easily for SO to supply hydrogen atoms to alkyl radicals (R**•**) than DM. While the hydrogen atoms provided by DM might be more easily combined with the SO radicals to restore SO to the original molecular structure. As shown in Figure 4A,B, the molecular structure of DM is more complex than that of SO, indicating that the steric hindrance of DM is greater than that of SO when reacting with free radicals on fatty acid chains. Therefore, it might be more easily for SO to react with alkyl radicals (R**·**) than DM, and DM was inclined to combine with the SO radicals.

When the DM factor was set aside in the design model of PBD, the selected model was still significant as the *F* value was 6.29. The regression coefficient obtained for *Y*(*k*) was 0.9041. The relative importance of other six parameters were presented as follows: F > B > E > D > G > C. F (SO), B (TP), and E (RA) also played key roles in inhibiting the oxidation of fish oil. Therefore, DM was considered to be omitted when other natural antioxidants was used in combination in this study.

### 3.3. Application of Synergist and Screened Natural Antioxidants in Anchovy Oil

**Figure 5:**
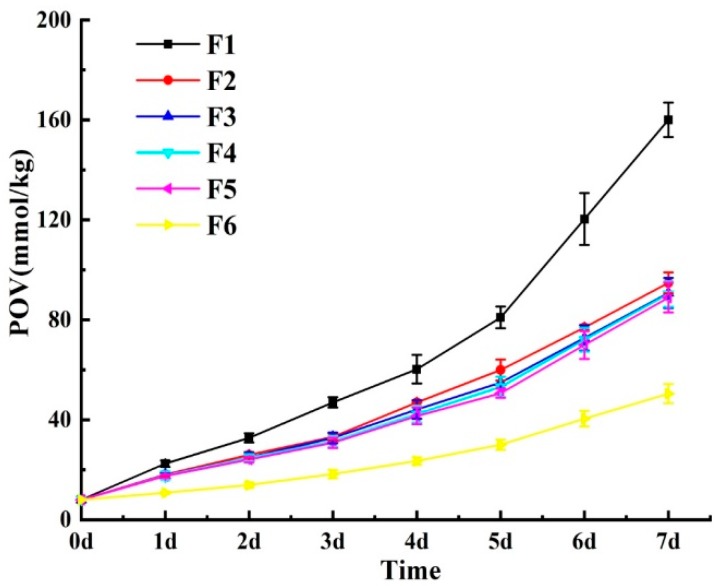
Peroxide value change of anchovy oils containing natural antioxidants or synergist during the storage experiment at 60 °C. Among them, F1 was fish oil without synergist or antioxidant. Data represent means ± S.D. for *n* = 3.

SO, TP, and RA presented good antioxidant ability in inhibiting the oxidation of anchovy oil when used in combination in this study. The combination of the three antioxidants was via application in anchovy oil, and then the antioxidant capacity of the combination was compared to TBHQ. Phytic acid and citric acid were two commonly used synergists. In order to compare their antioxidant properties when used in anchovy oil, these two synergists were used in combination with SO, respectively. The six experimental schemes described above were shown in Table 2. Storage experiments were carried out according to the experimental protocols. The POV of anchovy oils changes with storage time in these six experiments were shown in the Figure 5.

Sample F1 (Contrast) showed the fastest increase in POV among these six oil samples, increasing to 160.05 mmol/kg within seven days, which was 19.91 times of the initial value. Sample F6 (SO (0.04%) + phytic acid (0.2%)) showed the lowest increase in POV, increasing to 50.50 mmol/kg within seven days, which was 6.28 times higher than that the initial value. The POV of F2 (SO (0.04%)), F3 (TBHQ (0.02%)), F4 (SO (0.02%) + TP (0.02%) + RA (0.02%)) and F5 (SO (0.04%) + Citric acid (0.2%)) reached 94.83, 90.75, 90.33, and 88.94 mmol/kg after seven days, which were 11.79, 11.28, 11.23, and 11.06 times higher than the initial values, respectively.

The linear regression lines of these six samples during the seven days storage, the coefficients of determination (R^2^) and oxidation rate constants (*k*) were also calculated as shown in Table 5. The R^2^ of ten equations range from 0.9938 to 0.9994. The *k* of all these fish oil samples were showed as follows: *k* (F6) = 0.2589, *k* (F5) = 0.2678, *k* (F3) = 0.2691, *k* (F4) = 0.2694, *k* (F2) = 0.2758, and *k* (F1) = 0.3228. According to results from Figure 5 and Table 5, the antioxidant property of the screened antioxidant combination (SO (0.02%) + TP (0.02%) + RA (0.02%)) was similar to that of TBHQ. Both phytic acid and citric acid could enhance the antioxidant capacity of SO. Phytic acid owned chelating capacity of multivalent metallic ions, especially iron. It inhibited the iron-catalyzed hydroxyl radical formation and then blocking its coordination site [42,43]. Canan et al. [44] conducted the research on phytic acid showing that the purified phytic acid from rice bran had an iron ion chelating capacity based on the BPS (bathophenanthroline) method and the capacity was dependent on the concentration and contact time with the iron for the formation of the Fe^2+^–phytic acid complex. CA was one of the organic acids and used to adjust pH, also as a chelating agent to form complexes with multivalent metal ions and as dispersing agent to stabilize emulsions and multiphase systems [45]. Qiu et al. [46] reported that chitosan combined with citric acid or licorice extract could had a preserving effect on fresh fish fillets during frozen storage. Citric acid and licorice extract all could enhance the antioxidant ability of chitosan.

The addition of citric acid made the antioxidant properties of SO similar to TBHQ. The addition of phytic acid made antioxidant ability of SO superior to TBHQ, which indicated that phytic acid was more effective in increasing the antioxidant properties of SO. Phytic acid was called as myo-inositol hexaphosphoric acid and citric acid was called as 3’-hydroxy-3-biphenylcarboxylic acid. Their molecular structures were showed in Figure 4C,D. These molecular structure differences of these two synergists might lead to their ability differences of metal ions chelation, which might be the reason that phytic acid was more effective than citric acid in increasing the antioxidant properties of SO. When SO was added individually (F2), the antioxidant ability of SO was weaker than that of TBHQ. When SO was combined with phytic acid (F6), the antioxidant ability of the combination was superior to TBHQ. Therefore, phytic acid had a significant impact on the antioxidant ability of SO, which might be due to the high content of metal ions. The content of transition metal ion including Cr, Fe, Cu, and Zn in anchovy oil in this study was as high as 9.525 mg/kg, which could become an important factor affecting the oxidative stability of anchovy oil. Phytic acid was a good chelating agent for these metal ions, resulting in the combination of SO and phytic acid with better antioxidant ability than TBHQ.

## 4. Conclusions

The natural antioxidant combination suitable for anchovy oil was screened from the selected natural antioxidants through the application of PBD. The combination (SO (0.02%) +T P (0.02%) + RA (0.02%)) presented good antioxidant effect and its antioxidant ability was similar to that of TBHQ. When DM was used in combination with other antioxidants, its antioxidant effect was not as good as that of SO. However, DM had the best antioxidant effect among these several selected natural antioxidants when they were used alone, indicating that DM was suitable for use alone in this study. The combination of synergist (phytic acid or citric acid) and SO enhanced the antioxidant ability of SO used in anchovy oil. The synergistic effect of phytic acid was better than that of citric acid, and the antioxidant ability of the combination (SO + phytic acid) was better than that of TBHQ. This study provides a screening method of natural antioxidants combination from many antioxidants, and the combination could significantly affect the oxidation stability of anchovy oil. It also showed a reference for the industrial applications of antioxidants and synergists in anchovy oil. However, the optimal addition amount of the natural antioxidant combination for achieving the best antioxidant effect should be further studied.

## Figures and Tables

**Table 1:** Experimental variables at different levels and coded values using PBD design for screening of natural antioxidant.

Variables	Unit	Symbol	Coded Variables Levels	
			−1	1
X1: DM	%	A	0.01	0.02
X2: TP	%	B	0.02	0.04
X3: CA	%	C	0.02	0.04
X4: AP	%	D	0.01	0.02
X5: RA	%	E	0.02	0.04
X6: SO	%	F	0.02	0.04
X7: FA	%	G	0.01	0.02
X8, X9, X10, X11: Dummy factors	-	-	−1	1

DM: dihydromyricetin, TP: teapolyphenol, CA: carnosic acid, AP: ascorbyl palmitate, RA: rosemary acid, SO: sesamol, FA: ferulic acid.

**Table 2:** The antioxidants combination or the combination of antioxidants and synergists applied in anchovy oil.

NO	Synergist or Antioxidant (Additive Amount, w%)
F1	Contrast (anchovy oil sample without synergist or antioxidant)
F2	Sesamol (0.04%)
F3	TBHQ (0.02%)
F4	Sesamol (0.02%) + Teapolyphenol (0.02%) + Rosemary acid (0.02%)
F5	Sesamol (0.04%) + Citric acid (0.2%)
F6	Sesamol (0.04%) + Phytic acid (0.2%)

TBHQ: Tertiary butylhydroquinone.

**Table 3:** TOTOX value change of anchovy oils containing natural antioxidants during the storage experiment at 60 °C.

Antioxidants	Storage Time (Days)						
	1 **^A^	2 ^AB^	3 ^B^	4 ^C^	5 ^D^	6 ^E^	7 ^F^
Contrast *^C^	107.47 ± 1.96 ^f^	151.75 ± 8.37 ^e^	213.12 ± 1.91 ^f^	270.95 ± 8.29 ^g^	360.62 ± 2.13 ^h^	526.28 ± 5.48 ^f^	690.59 ± 12.49 ^g^
DM ^A^	52.03 ± 2.03 ^a^	56.85 ± 1.60 ^a^	61.68 ± 3.86 ^a^	66.63 ± 2.17 ^a^	73.76 ± 2.09 ^a^	83.02 ± 1.49 ^a^	92.28 ± 5.29 ^a^
TP ^B^	82.80 ± 0.68 ^bcd^	113.39 ± 0.71 ^bc^	143.53 ± 1.67 ^bc^	170.95 ± 2.37 ^b^	212.21 ± 0.47 ^b^	305.06 ± 0.85 ^b^	419.66 ± 7.78 ^b^
FA ^B^	98.60 ± 4.70 ^e^	111.96 ± 4.18 ^bc^	165.33 ± 1.11 ^d^	208.20 ± 4.30 ^de^	260.61 ± 1.97 ^e^	346.93 ± 1.22 ^d^	450.23 ± 0.21 ^cd^
VC ^B^	80.59 ± 3.79 ^bcd^	115.36 ± 2.46 ^bc^	160.76 ± 2.01 ^d^	204.90 ± 2.09 ^de^	269.93 ± 1.51 ^f^	366.89 ± 15.96 ^e^	484.08 ± 2.95 ^ef^
SO ^B^	86.76 ± 2.37 ^d^	120.43 ± 4.68 ^c^	151.62 ± 6.19 ^c^	210.76 ± 0.45 ^e^	268.05 ± 0.95 ^ef^	342.13 ± 6.01 ^d^	418.08 ± 1.21 ^b^
LE ^B^	96.61 ± 1.59 ^e^	136.74 ± 6.66 ^d^	181.34 ± 0.94 ^e^	232.03 ± 1.02 ^f^	295.77 ± 4.32 ^g^	374.10 ± 1.88 ^e^	498.92 ± 1.46 ^f^
RA ^B^	82.38 ± 3.05 ^cd^	111.15 ± 2.16 ^bc^	162.64 ± 1.66 ^d^	206.18 ± 1.66 ^de^	262.83 ± 0.20 ^ef^	335.94 ± 3.23 ^d^	468.39 ± 4.47 ^de^
AP ^B^	76.86 ± 1.33 ^bc^	112.59 ± 3.78 ^bc^	151.01 ± 1.23 ^c^	199.04 ± 1.18 ^cd^	245.17 ± 3.04 ^d^	327.98 ± 1.07 ^cd^	437.60 ± 0.26 ^bc^
CA ^B^	73.16 ± 3.34 ^b^	101.54 ± 1.09 ^b^	135.73 ± 0.75 ^b^	190.62 ± 1.81 ^c^	233.56 ± 3.42 ^c^	312.40 ± 2.43 ^bc^	417.25 ± 16.85 ^b^

* Contrast: fish oil without natural antioxidant. ** Values are the mean ± S.D. for *n* = 3. ^a–h^ Samples with the same letter in a column are not significantly different (*p* < 0.05) according to one-way ANOVA analysis. ^A–F^ Samples with the same letter are not significantly different (*p* < 0.05) according to two-way ANOVA analysis with the storage time and antioxidant as factors, and TOTOX as dependent variable.

**Table 4:** The first-order reaction rate equations for oxidation kinetics of anchovy oils added with individual antioxidant and their coefficients of determination (*R*^2^), oxidation rate constants (*k*).

Antioxidants	Equations	*R* ^2^	*k*
Contrast	*Y*^a^ = 0.3228*X* ^b^ + 0.7397	0.9972	0.3228
DM	*Y* = 0.1087*X* + 0.0465	0.9914	0.1087
TP	*Y* = 0.2727*X* + 0.5123	0.9906	0.2727
FA	*Y* = 0.2704*X* + 0.6419	0.9927	0.2704
VC	*Y* = 0.3137*X* + 0.4451	0.996	0.3137
SO	*Y* = 0.2758*X* + 0.5977	0.9938	0.2758
LE	*Y* = 0.2814*X* + 0.7005	0.9936	0.2814
RA	*Y* = 0.2974*X* + 0.5019	0.9928	0.2974
AP	*Y* = 0.2986*X* + 0.4548	0.9933	0.2986
CA	*Y* = 0.3072*X* + 0.3573	0.9948	0.3072

*Y*^a^ stands for ln (POV). *X* ^b^ stands for storage time (days).

**Table 5:** The reaction rate equations of anchovy oils oxidation for antioxidants or synergist application and their coefficients of determination (*R*^2^), oxidation rate constants (*k*).

NO	Equations	*R* ^2^	*k*
F1	*Y* = 0.3228*X* + 0.7397	0.9972	0.3228
F2	*Y* = 0.2758*X* + 0.5977	0.9938	0.2758
F3	*Y* = 0.2691*X* + 0.5782	0.9965	0.2691
F4	*Y* = 0.2694*X* + 0.5569	0.998	0.2694
F5	*Y* = 0.2678*X* + 0.5388	0.9979	0.2678
F6	*Y* = 0.2589*X* + 0.0378	0.9994	0.2589

## Supplementary Materials

The following are available online at https://www.mdpi.com/2076-3921/8/12/627/s1, Table S1: Experimental design using the PBD for the screening of antioxidants significantly effecting the oxidative stability of anchovy oil; Table S2: The reaction rate equations of anchovy oils oxidation kinetics for PBD and their coefficients of determination (*R*^2^), oxidation rate constants (k); Table S3: Regression coefficients and corresponding F and p values for k in seven variable PBD design experiment.
