# Supplementary material for: Application of Plackett–Burman Design in Screening of Natural Antioxidants Suitable for Anchovy Oil
Source: Antioxidants (Basel). 2019 Dec 6;8(12):627. doi: 10.3390/antiox8120627 (PMC6943644; doi:10.3390/antiox8120627)
Supplement: Supplementary file 1 [file antioxidants-08-00627-s001.pdf]

Table S1. Experimental design using the PBD for the screening of antioxidants significantly effecting the oxidative stability of anchovy oil.

Run	Coded value							Y(*k*)
	A	B	C	D	E	F	G	
1	-1	1	-1	1	1	-1	1	0.1220
2	-1	1	1	-1	1	1	1	0.1179
3	1	1	-1	-1	-1	1	-1	0.1164
4	-1	-1	-1	-1	-1	-1	-1	0.1323
5	1	-1	1	1	-1	1	1	0.1236
6	-1	-1	-1	1	-1	1	1	0.1261
7	1	1	-1	1	1	1	-1	0.1145
8	1	1	1	-1	-1	-1	1	0.1201
9	1	-1	-1	-1	1	-1	1	0.1242
10	1	-1	1	1	1	-1	-1	0.1241
11	-1	-1	1	-1	1	1	-1	0.1216
12	-1	1	1	1	-1	-1	-1	0.1327

Table S2. The reaction rate equations of anchovy oils oxidation kinetics for PBD and their coefficients of determination (R^2), oxidation rate constants (*k*)

Run	Eq.	R^2	*k*
1	y=0.122x+0.0787	0.9900	0.1220
2	y=0.1179x+0.0405	0.9999	0.1179
3	y=0.1164x-0.0441	0.9946	0.1164
4	y=0.1323x+0.0881	0.9844	0.1323
5	y=0.1236x-0.055	0.9964	0.1236
6	y=0.1261x+0.0316	0.9999	0.1261
7	y=0.1145x+0.0391	0.9991	0.1145
8	y=0.1201x+0.1534	0.9919	0.1201
9	y=0.1242x+0.0628	0.9984	0.1242
10	y=0.1241x+0.0899	0.9916	0.1241
11	y=0.1216x+0.1897	0.9908	0.1216
12	y=0.1327x+0.0687	0.9902	0.1327

Table S3. Regression coefficients and corresponding F and p values for k in seven variable PBD design experiment.

Source	Sum of Squares	df	Mean Square	F Value	p-value (Prob> F)	significant
Model	0.000320206	7	4.57437E-05	6.99	0.0395	*
A	7.35075E-05	1	7.35075E-05	11.24	0.0285	*
B	6.67408E-05	1	6.67408E-05	10.20	0.0331	*
C	1.6875E-06	1	1.6875E-06	0.26	0.6382	
D	9.1875E-06	1	9.1875E-06	1.40	0.3015	
E	6.03008E-05	1	6.03008E-05	9.22	0.0385	*
F	0.000103841	1	0.000103841	15.88	0.0163	*
G	4.94083E-06	1	4.94083E-06	0.76	0.4338	
Residual	2.61633E-05	4	6.54083E-06			
Cor Total	0.000346369	11				

*$p<0.05$.
